# Genetic stabilization of attenuated oral vaccines against poliovirus types 1 and 3

**DOI:** 10.1038/s41586-023-06212-3

**Published:** 2023-06-14

**Authors:** Ming Te Yeh, Matthew Smith, Sarah Carlyle, Jennifer L. Konopka-Anstadt, Cara C. Burns, John Konz, Raul Andino, Andrew Macadam

**Affiliations:** 1 Department of Microbiology and Immunology, University of California, San Francisco, San Francisco, CA USA; 2 National Institute for Biological Standards and Control, South Mimms, UK; 3 Division of Viral Diseases, Centers for Disease Control and Prevention, Atlanta, GA USA; 4 Center for Vaccine Innovation and Access, PATH, Seattle, WA USA

**Keywords:** Virology, Vaccines

## Abstract

Vaccination with Sabin, a live attenuated oral polio vaccine (OPV), results in robust intestinal and humoral immunity and has been key to controlling poliomyelitis. As with any RNA virus, OPV evolves rapidly to lose attenuating determinants critical to the reacquisition of virulence^1–3^ resulting in vaccine-derived, virulent poliovirus variants. Circulation of these variants within underimmunized populations leads to further evolution of circulating, vaccine-derived poliovirus with higher transmission capacity, representing a significant risk of polio re-emergence. A new type 2 OPV (nOPV2), with promising clinical data on genetic stability and immunogenicity, recently received authorization from the World Health Organization for use in response to circulating, vaccine-derived poliovirus outbreaks. Here we report the development of two additional live attenuated vaccine candidates against type 1 and 3 polioviruses. The candidates were generated by replacing the capsid coding region of nOPV2 with that from Sabin 1 or 3. These chimeric viruses show growth phenotypes similar to nOPV2 and immunogenicity comparable to their parental Sabin strains, but are more attenuated. Our experiments in mice and deep sequencing analysis confirmed that the candidates remain attenuated and preserve all the documented nOPV2 characteristics concerning genetic stability following accelerated virus evolution. Importantly, these vaccine candidates are highly immunogenic in mice as monovalent and multivalent formulations and may contribute to poliovirus eradication.

## Abstract (web summary)

Mouse experiments and deep sequencing confirmed that two additional live attenuated vaccine candidates against type 1 and 3 polioviruses remained attenuated and preserved all documented nOPV2 characteristics concerning genetic stability following accelerated virus evolution.

## Main

Three poliovirus serotypes exist. Wild poliovirus (WPV) types 2 and 3 have been eradicated but WPV1 still causes disease in Afghanistan and Pakistan^4,5^. Live attenuated oral poliovirus vaccine (OPV) and inactivated poliovirus vaccine (IPV) protect against polio. In many countries, a combination of bivalent OPV (bOPV, types 1 and 3) and IPV (types 1, 2 and 3) is routinely used. OPV is cheaper than IPV, it replicates in the gut, elicits better primary intestinal immunity than IPV and more effectively prevents poliovirus transmission. OPV indirectly immunizes unvaccinated persons with viruses shed by vaccinees^6–8^. Also, OPV is easily administered as oral drops and has been central to outbreak responses and efforts to end poliovirus.

Eradication of polioviruses is within reach^9^. If the objectives of the Global Polio Eradication Initiative are achieved—halting circulation of wild-type 1 poliovirus and vaccine-derived polioviruses (cVDPV)—routine use of bivalent OPV (types 1 and 3) will be ended. After that, the risk of vaccine-derived type 1 and 3 viruses will have to be managed in an environment where intestinal immunity, which prevents virus spread, drops over time. From 2016 to 2021, 273 WPV1 and 1,818 cVDPV cases were reported: 83 cVDPV1, 1,728 cVDPV2, six cVDVP3 and one recombinant of cVDPV2 and cVDPV3 (ref. ^10^). Although cases of cVDPV1 and 3 were fewer than those of cVDP2, all three strains were detected in environmental samples, posing a risk of spreading virulent viruses to other countries. cVDPV2 detections exploded in the years after cessation of routine use, peaking with 1,082 acute flaccid paralysis cases in 2020 (ref. ^11^). The vaccine used to control the outbreaks, Sabin monovalent OPV2, routinely seeded new outbreaks with increasing geographical spread due to serial transmission in susceptible populations^12^. Whereas most cases have been in Afghanistan, Pakistan and Africa, IPV-utilizing high-income countries—including the United States, United Kingdom and Israel—have all experienced cVDPV2 outbreaks from importations^13–15^.

To confront the challenge of reversion of OPVs to cVDPVs, we developed a safer type 2 vaccine strain (nOPV2). In brief, an RNA structure within the 5′ untranslated region (5′UTR) involved in attenuation of virus replication in neurons—domain V—was stabilized by replacement of all G–U pairs with C–G or U–A pairs so that the virus could not regain neurovirulence via a single point mutation^16–18^. The nOPV2 design also includes relocation of the *cis*-acting replication element (*cre*) to the 5′UTR, to protect modified domain V from replacement through a single recombination event, and two amino acid substitutions in the RNA polymerase (3D) to improve fidelity and reduce the frequency of recombination events^18^. Promising human safety, immunogenicity, viral shedding and genetic stability results from phase 1 and 2 studies of nOPV2 were recently reported^19–23^, leading to the first-ever vaccine Emergency Use Listing from the WHO (World Health Organization)^24^. Since March 2021, more than 600 million doses of nOPV2 have been administered in outbreak responses in 23 countries, with a promising decline in cVDPV2 cases in both 2021 and 2022 (ref. ^11^) and early evidence of effectiveness in halting ongoing outbreaks^25,26^.

Highly attenuated and genetically stable type 1 and 3 vaccine candidates, such as nOPV2, could be used in an outbreak response. Acceleration of the licensure and WHO prequalification of nOPV2 may facilitate wider use of the vaccine^26^: it may be better accepted due to the perception that nOPVs are safer than previous Sabin viruses. On the basis of the superior stability of attenuation for nOPV2 (refs. ^18,19,22,23,27–30^), we developed new OPV vaccine candidates for type 1 and 3 polioviruses by replacing the capsid-coding region of nOPV2 with that of Sabin 1 and 3. These candidates preserve the antigenic and immunogenic characteristics of Sabin 1 and 3 and increase their safety by stabilizing determinants of attenuation. These features could lead to safer monovalent type 1 and 3 and multivalent OPVs. To prevent reoccurrence of the epidemiological conditions after OPV2 cessation, the more stable type 1 and 3 OPVs must be accessible before normal cessation of bivalent OPVs.

## Engineering Sabin 1 and 3 genomes

**Fig. 1:**
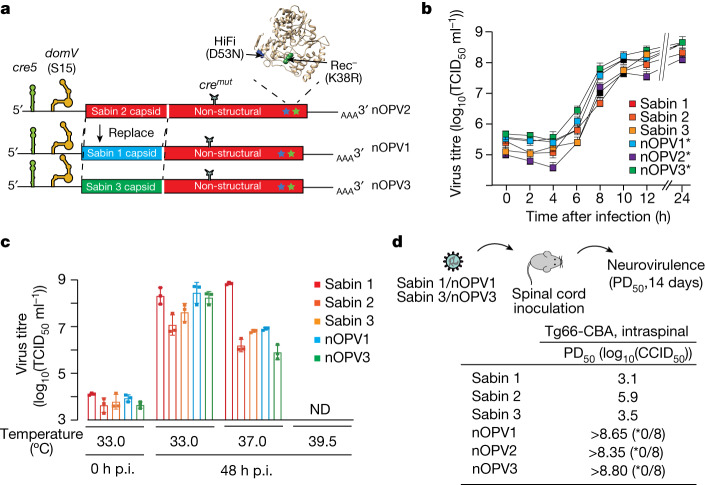
Characterization of nOPVs. **a**, Schematic representation of the predicted RNA secondary structures of the relocated *cis-*acting replication element (*cre5*); predicted RNA secondary structure of *domV* carrying mutated bases (for sequence details see Extended Data Fig. 1a) to stabilize the structure; the recoded, inactivated 2C-*cre* (*cre*^*mut*^); and the fidelity (D53N) and recombination (K38R) determinant in the viral 3D RNA polymerase of nOPV2. The Sabin 2 capsid-coding region is replaced by Sabin 1 (blue) and Sabin 3 (green) capsid-coding regions to generate nOPV1 and nOPV3, respectively. **b**, One-step replication analysis of the modified viruses. Asterisks denote viruses carrying all nOPV2 modifications except *Rec*. Data are mean ± s.d. of triplicates (*n* = 3 samples). **c**, Virus production and temperature susceptibility. Vero cells were infected at MOI = 0.01 and collected after incubation for 48 h at the indicated temperatures. Standard TCID_50_ was performed to determine virus titres. Data shown as mean ± s.d. of triplicates (*n* = 3 samples). h.p.i., hours post incubation; ND, not detected (that is,no cytopathic effect was observed from the TCID_50_ assay). **d**, Top, schematic representation of neurovirulence test. Bottom, PD_50_ values for Sabin and nOPV viruses were determined by intraspinal inoculation in transgenic mice expressing the human poliovirus receptor (Tg66-CBA). Asterisk denotes the paralysed/total at the highest dose. Source Data

**Extended Data Fig. 1:**
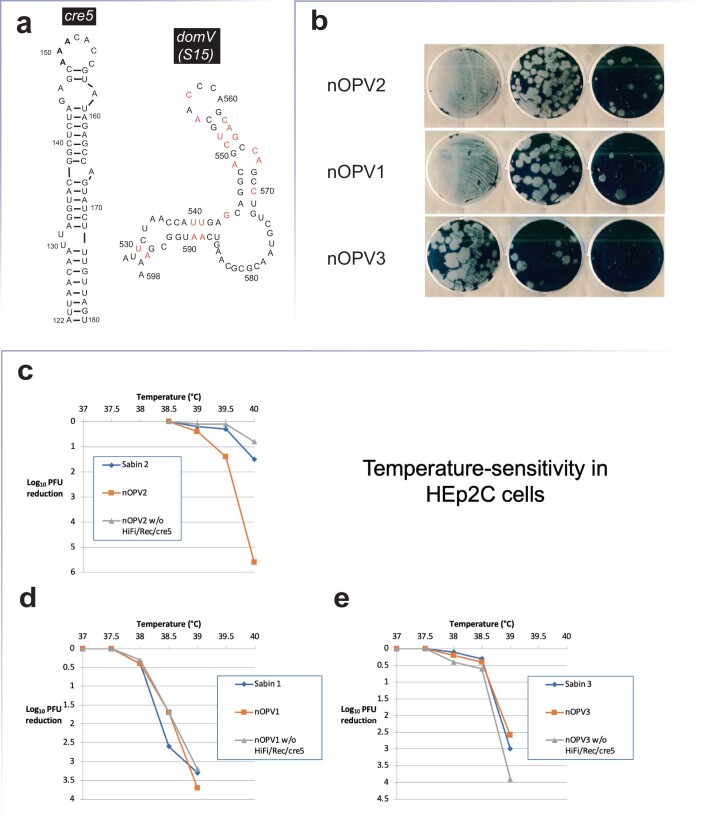
Growth characterization of the nOPVs. (**a**) Schematic representation of the predicted RNA secondary structures of the relocated *cis-*acting replication element (cre5); predicted RNA secondary structure of the domV and the mutated bases (shown in red) introduced to stabilize the structure; (**b**) Plaque morphology of the nOPVs. (**c–e**) Temperature susceptibility of the types 2 (**c**), 1 (**d**) and 3 (**e**) nOPVs. Blue-diamond, Sabin virus; orange-square, nOPV; grey upper-triangle, nOPV without HiFi/Rec or cre relocation. Source Data

To engineer new type 1 and 3 Sabin strains, we replaced the capsid (P1) region of nOPV2 candidate 1 (-c1) with the capsid region from a Sabin 1 and Sabin 3 clone to generate nOPV1-c1 and nOPV3-c1, respectively (Fig. 1a). For simplicity, the -c1 designation is omitted hereafter. This strategy preserved all five modifications introduced in nOPV2 (that is, two modifications within the 5′UTR—relocated *cre* and S15 *domV*), synonymous mutations at eight nucleotide positions in the 2C coding region to inactivate the internal *cre*^31^ and two mutations in the 3D polymerase (D53N and K38R) to limit viral adaptability (Fig. 1a and Extended Data Fig. 1a). The capsid regions of the Sabin strains all contain attenuating mutations: A4065S (a change from alanine to serine at VP4 position 65), L3225M, A1106T and L1134F for Sabin 1; T1143I for Sabin 2; and S3091F and I1006T for Sabin 3 (refs. ^3,32–34^).

Because any live attenuated vaccine must be able to multiply sufficiently for production, we examined the multiplication properties of nOPV1 and nOPV3 with all the above modifications (Fig. 1a) in Vero cells used for vaccine production^35^. Plaque size phenotypes of nOPV1 and nOPV3 were similar to those of nOPV2 (Extended Data Fig. 1b). In addition, replication fitness was not reduced as shown by one-step replication analysis (Fig. 1b). In Vero cells at 33 °C, nOPV1 and nOPV3 replicated to comparable or higher titres than Sabin 1 and 3 with similar replication kinetics, and they maintained their temperature sensitivity at 37.0 and 39.5 °C similar to Sabin 2 (Fig. 1c).

Each modification in nOPV1 and nOPV3 (*cre5*, S15 *domV*, HiFi(D53N) and Rec1(K38R)) contributes independently to attenuation^18^, and their combination in the same virus creates a multilayer safety net that reduces the likelihood of regaining fitness and neurovirulence. To examine the effects of these substitutions we initially used a cell culture assay that estimates temperature sensitivity, which is a characteristic correlating with attenuation^1,17^. Replacing *domV* with S15 had little or no effect on Sabin 2 sensitivity at the tested temperatures (Extended Data Fig. 1c), whereas introduction of 3D polymerase modifications and the *cre5* relocation produced viruses that were more temperature sensitive than Sabin 2 (Extended Data Fig. 1c). By contrast, nOPV1 and nOPV3 viruses showed thermosensitivity similar to their counterparts Sabin 1 and 3 with or without the *cre5* relocation and 3D polymerase modifications (Extended Data Fig. 1d,e).

We determined the median paralysis dose (PD_50_) for each mutant virus in a transgenic mouse neurovirulence test^36^ as used with new lots of Sabin vaccine (Methods). PD_50_ values (in log_10_(50% tissue culture infectious dose (TCID_50_)) units) were determined as 3.1, 5.9 and 3.9 for Sabin 1, 2 and 3, respectively. All three new vaccine strains were more attenuated than Sabin 1, 2 and 3, with PD_50_ values over 8.0 (Fig. 1d).

**Extended Data Fig. 2:**
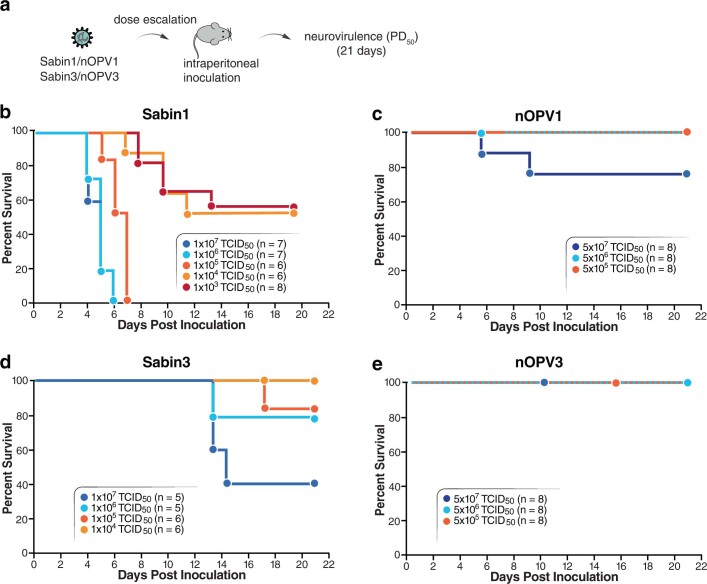
Virulence of the Sabin 1, nOPV1, Sabin 3 and nOPV3. Ten-day-old Tg21/IFNR^-/-^ mice were i.p. inoculated (a) with 100 μL of inoculum delivering various amounts of Sabin 1 (b), nOPV1(c), Sabin 3 (d), or nOPV3 (e) and monitored for 21 days. Source Data

To further examine neuroinvasion and neurovirulence, we inoculated susceptible mice intraperitoneally with various doses of nOPV1, nOPV3 and Sabin 1 and 3 (Extended Data Fig. 2a). Following inoculation with the higher doses no mice inoculated with Sabin 1 survived (1 × 10^5^−1 × 10^7^ TCID_50_), and 60% of the mice inoculated with Sabin 3 (1 × 10^7^ TCID_50_) were paralysed (Extended Data Fig. 2b,d). By contrast, survival percentages were 75 and 100 for mice inoculated with the highest dose (5 × 10^7^ TCID_50_) of nOPV1 and nOPV3, respectively (Extended Data Fig. 2c,e). This result is consistent with those from the above-mentioned neurovirulence test in Tg66 mice (TgmNVT), and thus we concluded that nOPV1 and nOPV3 are more attenuated than the original Sabin 1 and 3.

## Genetic stability of nOPV1 and nOPV3

**Fig. 2:**
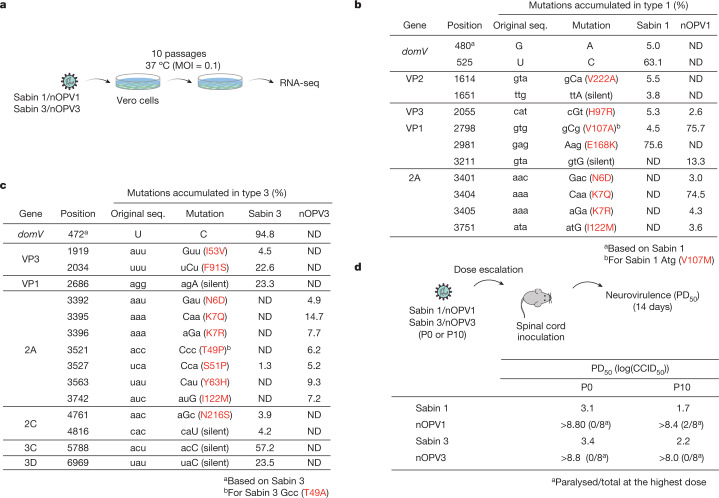
Genetic stability of nOPVs. **a**, Experimental design of the genetic stability test. **b**,**c**, Sabin 1 and nOPV1 (**b**) or Sabin 3 and nOPV3 (**c**) were used to infect Vero cells at MOI = 0.1 and grown at 37 °C for 10 h. After ten rounds of accelerated evolution the virus population was sequenced and analysed with LowFreq. Mutations with frequencies greater than 3% are listed; those not called are denoted by ND. Data are from one experiment. Uppercase letters indicate mutated codon position. Original seq. refers to the sequence of the infectious cDNA clone. **d**, Genetic stability was also examined by a neurovirulence test. PD_50_ values of the original virus population (P0) and at passage 10 (P10) were determined by infection of Tg66 mice via intraspinal inoculation. Source Data

Next we determined whether nOPV1 and nOPV3 are genetically more stable than the current Sabin 1 and 3. Initially we used a simple cell culture evolution paradigm (Fig. 2a). The thermosensitive phenotypes of Sabin 1 and 3 are more pronounced in certain cell lines. For example, serial passage of Sabin viruses at 37 °C in Vero cells, but not in Hep2C, promotes the rapid incorporation of mutations that increase replicative fitness and thermotolerance^1^. These thermoresistant variants also show a significant increase in virulence^1,17^.

**Fig. 3:**
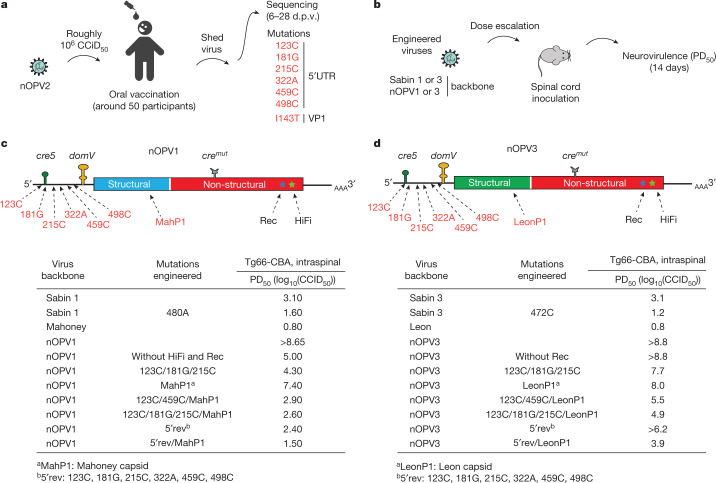
Evaluation of potential neurovirulent strains by engineering mutations into nOPV1 and nOPV3 that accumulate in nOPV2-vaccinated humans. **a**, The most frequent mutations identified from viruses shed from nOPV2-vaccinated humans. Known virulence factors *domV* position 480A (Sabin 1) and 472C (Sabin 3) are included for comparison. **b**, Virulence (PD_50_) of reconstructed viruses was determined with the Tg66-CBA mouse model following intraspinal inoculation. **c**,**d**, In addition, the Sabin capsid coding region was replaced with that derived from neurovirulent Mahoney (MahP1) (**c**) or Leon (LeonP1) (**d**). d.p.v., days post vaccination. Source Data

**Extended Data Fig. 3:**
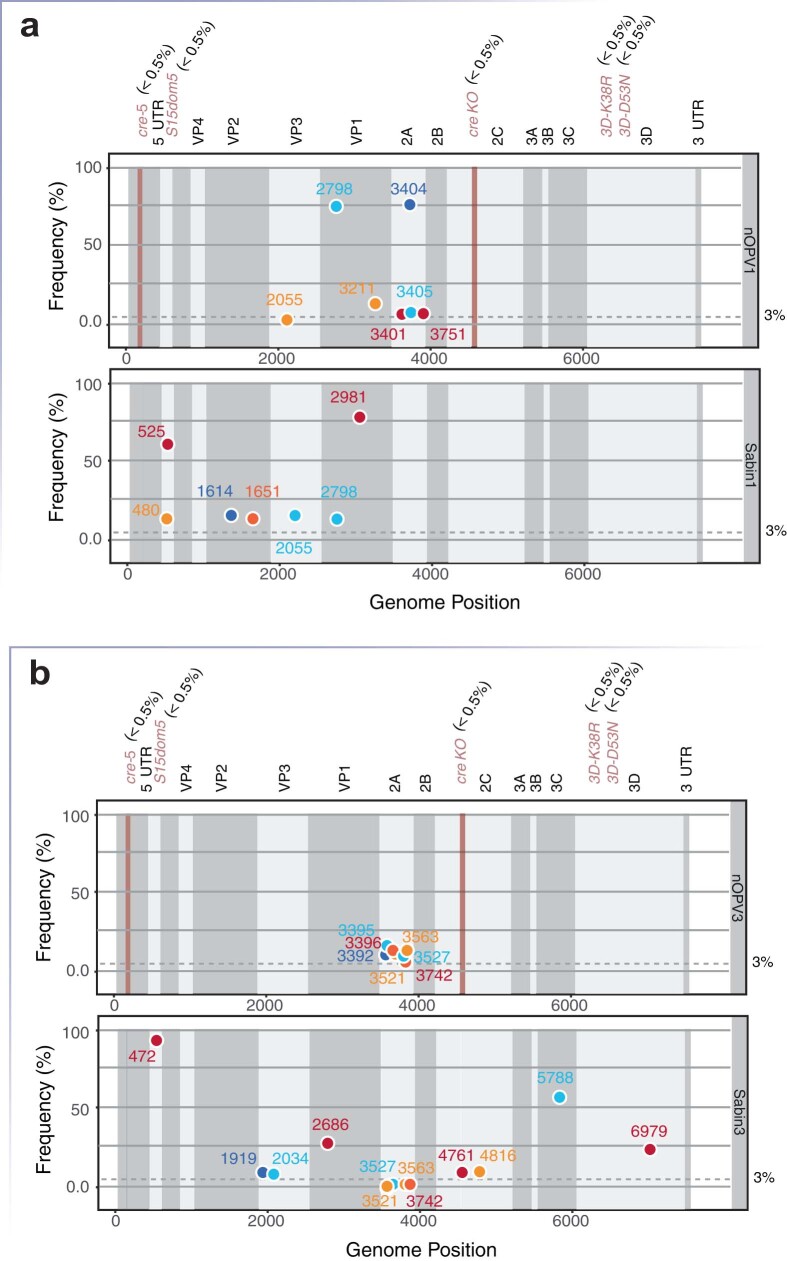
Genetic stability of the viruses after accelerated evolution. Manhattan plot showing the frequency of mutations at several locations, including the 5′-*cre*, *domV*, 2C*cre*^*mut*^, and *HIFi/Rec* in (**a**) Sabin 1 (upper) and nOPV1 (lower) and (**b**) Sabin 3 (upper) and nOPV3 (lower).

We carried out serial passage experiments with large viral population sizes (10^6^ plaque-forming units) and low multiplicity of infection (MOI = 0.1) that further ensured rapid evolution^37^. After ten passages we used next-generation sequencing to determine the variant frequency of populations of nOPV1 and nOPV3 and parental Sabin 1 and 3. Whereas control Sabin vaccine strains acquired the neurovirulence determinants within *domV*, nucleotides 480 and 525 (5 and 63%, respectively, for Sabin 1; Fig. 2b and Extended Data Fig. 3a) and 472 (about 95% for Sabin 3; Fig. 2c and Extended Data Fig. 3b), no mutations with frequency greater than 1% were identified within *domV*, nor for the four other modifications introduced into nOPV2 strains (Fig. 3). However, mutations accumulated—mostly in the 2A region—at frequencies of 4–75% in both Sabin strains and nOPV1 and nOPV3 populations (Fig. 2b,c and Extended Data Fig. 3a,b). These 2A mutations are associated with cell culture adaptation and have no effect on monkey neurovirulence^38^. We also found substitutions in VP1 and VP3 and silent mutations in Sabin and nOPV strains (Fig. 2b,c). However, a review of the existing literature showed no evidence that any of these mutations have an effect on virulence.

We next examined the virulence of nOPV1 and nOPV3 by direct intraspinal inoculation of transgenic mice expressing the human poliovirus receptor^36^. PD_50_ values for Sabin 1 and 3 in this model were determined as 3.1 and 3.9 log_10_(50% cell culture infective dose (CCID_50_)), respectively (Fig. 2d; P0). Notably, nOPV1 and nOPV3 induced no paralysis in any mouse even at the highest available dose, up to approximately 8.8 log_10_(CCID_50_), indicating significantly lower neurovirulence than the Sabin strains. We also examined the neurovirulence of populations of Sabin and nOPV vaccine candidates following high-temperature growth selection in Vero cells (Fig. 2d; P10). In agreement with the observation that Sabin 1 and 3 accumulated mutations within *domV*, high-temperature-adapted Sabin 1 and 3 viruses exhibited reduced PD_50_ values of 2.2 and 1.7 log_10_(CCID_50_), respectively. After ten passages, the nOPV1 strain caused paralysis in two mice at the highest inoculated dose of 8.4 log_10_(CCID_50_), which may represent a small increase in neurovirulence compared with P0, but the strain was still significantly more attenuated than the original Sabin 1 (Fig. 2d; P10). On the other hand, nOPV3 remained attenuated after ten passages with no measurable neurovirulence at the highest tested dose (Fig. 2d). Although clinical trials are required for full evaluation of the genetic stability of vaccine candidates after replication in humans, our deep sequencing and mouse neurovirulence testing confirmed that the designed vaccine candidates nOPV1 and nOPV3 are more resistant to reversion to neurovirulence than the Sabin strains.

## Vaccinee-isolated mutations on virulence

Several common mutations have previously been identified among isolates recovered from vaccinees with nOPV2. To determine the effects of these mutations in virus shed from vaccinees we engineered each of them, in different combinations, into the infectious complementary DNA clones of nOPV1 and nOPV3 and analysed their effects on virus fitness and virulence. We introduced single or multiple substitutions 123C and 181G, 215C, 322A, 459C and 498C. We replaced the capsid coding region with that derived from wild-type Mahoney type 1 and Leon type 3. We also generated nOPV1 and nOPV3 carrying six mutations within the 5′ UTR (*5*′*rev*) (Fig. 3). Furthermore, we combined some 5′ UTR mutations with the wild-type capsid replacement. Although we did not attempt to analyse all possible combinations or evolutionary trajectories, this extensive analysis provided a rich set of information that can be useful in evaluation of the probability of nOPV1 and nOPV3 evolving into neurovirulent forms following circulation in humans (Fig. 3). For example, the reconstructed *5*′*rev/MahP1* and *5*′*rev/LeonP1*, containing all 5′ UTR mutations and replacement of the P1 region, represent the ‘worst-case’ scenario. Many of the mutations selected in shed viruses after many weeks of replication had accumulated to 100% in a single genome, imparting the maximum increase in fitness^19,39^. For comparison we used Sabin 1/480A and Sabin 3/472C, which are the de facto cases when Sabin 1 and 3 are used (that is, a revertant virus excreted by most vaccinees within 1–2 weeks of vaccination). When neurovirulence was evaluated by intraspinal inoculation of susceptible mice, both 480A in Sabin 1 and 472C in Sabin 3 produced neurovirulent viruses, with PD_50_ values of 1.6 and 1.2 (log_10_(TCID_50_)), respectively. The mutant *5*′*rev/MahP1* and *5*′*rev/LeonP1* yielded PD_50_ values of 1.5 and 3.9, respectively. Thus, even in the unlikely event that nOPV3 accumulated all changes in one genome, the new strain would be around 400 times more attenuated than the single-mutation Sabin 3 revertant (Sabin 3/472C), which is produced almost universally days after vaccination^33^. For Sabin 1 we find that the acquisition of all observed mutations will render a virus that is similar to reverted Sabin 1/480A but, to acquire all observed mutations, the virus would need to acquire six independent 5′ UTR mutations and multiple mutations in the P1 region. Importantly, all intermediate viruses with fewer mutations are much less virulent than Sabin strains with a single mutation within *domV* (Fig. 3).

## Antigenicity and immunogenicity analysis

**Extended Data Fig. 4:**
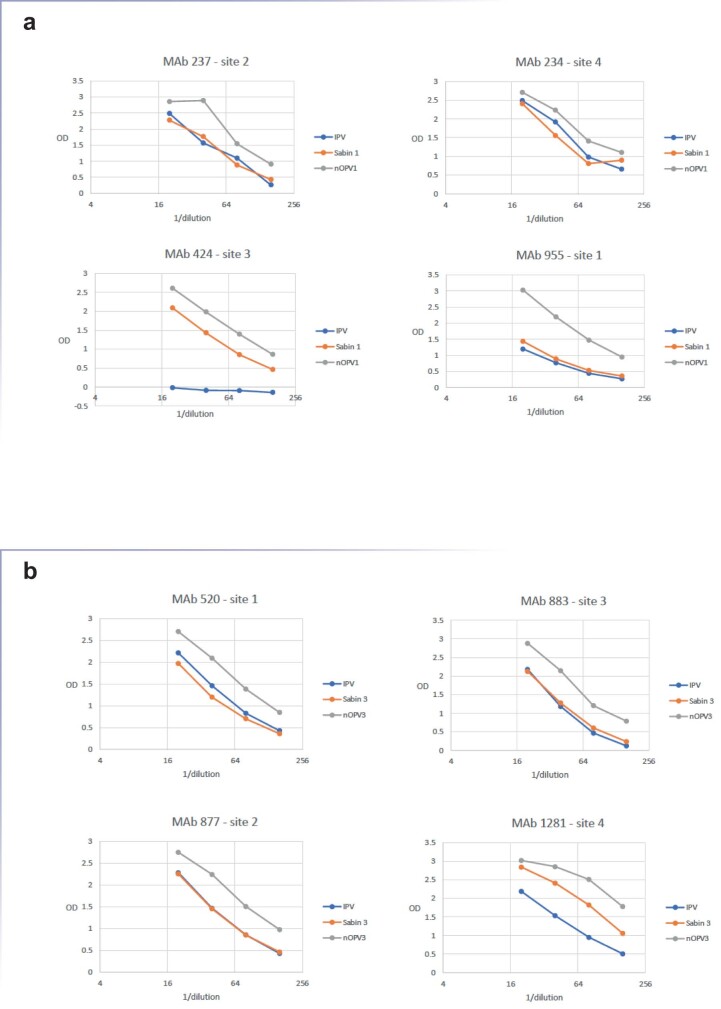
Antigenicity of Sabin 1/3 and nOPV 1/3. Reactivities of (**a**) Sabin1 (upper) and nOPV1 (lower), and (**b**) Sabin 3 (upper) and nOPV3 (lower) with monoclonal antibodies against four antigenic sites on the poliovirus type 1 or type 3 virion were compared in ELISA assays. Source Data

Because the nOPV1 and nOPV3 strains are intended to elicit immune protection against virulent poliovirus strains, we analysed the antigenic structure of the vaccine candidate in an ELISA assay similar to that used to measure the D-antigen content of poliovirus vaccines. A panel of monoclonal antibodies, specific for Sabin 1 native conformations of antigenic sites 1 (antibody 955), 2 (237), 3 (424) and 4 (234), were used as primary antibodies^40^. We also examined Sabin 3 native conformations of antigenic sites using monoclonal antibodiess directed against site 1 (antibody 520), 2 (877), 3 (883) and 4 (1,281)^40^. The Sabin 1 and 3 strains and IPV1 and IPV3 were used as a reference in each experiment. Differences in antigenicity from Sabin references were not noted at any of the four sites tested for type 1 and 3 polioviruses (Extended Data Fig. 4a,b).

**Fig. 4:**
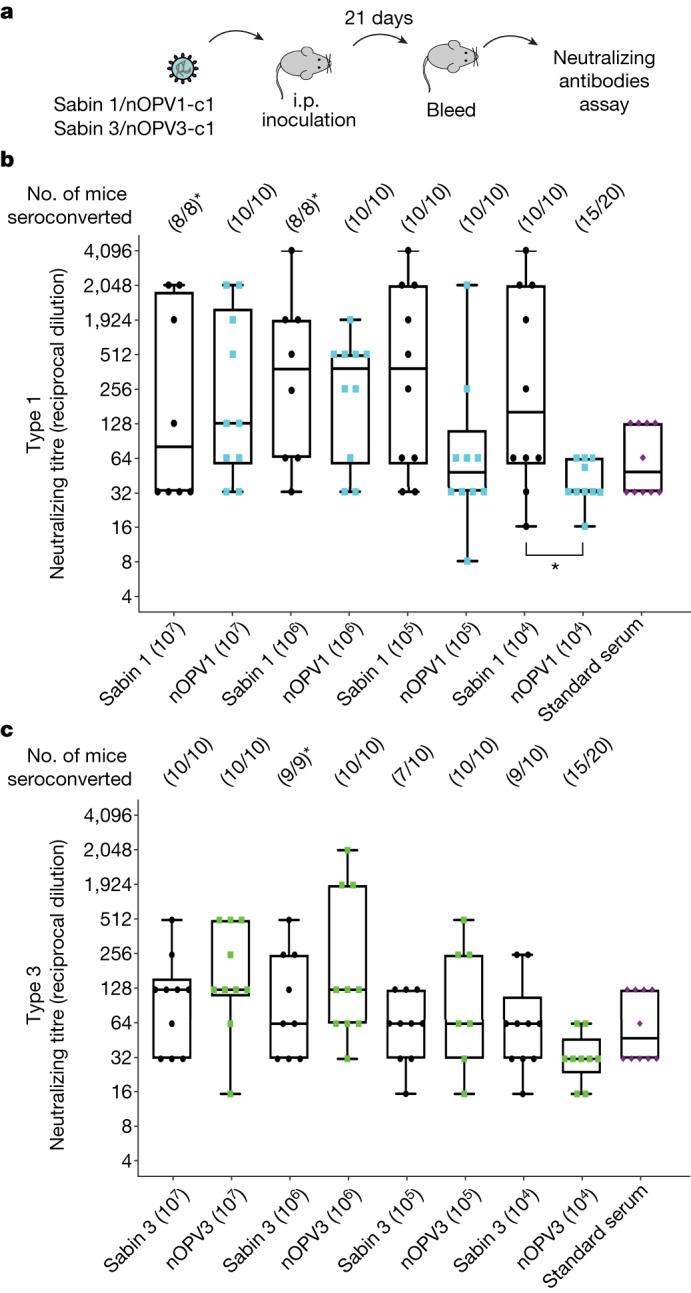
Immunogenicity of nOPV1 and nOPV3 vaccine candidates in mice. **a**, PVRTg21 with type  I interference receptor knockout (PVRTg21/IFNR-KO) mice were inoculated intraperitoneally (i.p.) with doses (10^4^–10^7^ TCID_50_) of Sabin 1 and 3 vaccine viruses or nOPV1 and nOPV3 vaccine candidates. Ten mice were used per condition. Titres of neutralizing antibodies in sera at day 21 were determined by neutralization assay (Methods). **b**,**c**, Data corresponding to type 1 (Sabin 1 and nOPV1) (**b**) or type 3 (Sabin 3 and nOPV3) (**c**). **b**,**c**, Box-and-whisker diagrams represent neutralizing antibody response for each condition; bars in boxes represent median antibody titres; whiskers range from minimum to maximum, with all non-outliers (less than 2.5× interquartile range from the median) shown. Outlying points are plotted individually. Overlapping dots represent neutralizing antibody values obtained for each mouse. The two-sided Mann–Whitney *U*-test detected a significant difference only at the lowest titre (10^4^ TCID_50_) of Sabin 1 and nOPV1 (*P* = 0.0225) (*n* = 8 or 10 (**b**) and *n* = 10 (**c**) samples); no significance was observed at other titres. Standard serum is serum from a human participant vaccinated with OPV. Seroconversion frequency (number of individuals that seroconverted from the total) is shown. Asterisks indicate that some mice inoculated with Sabin viruses died and were removed from the experiment. Source Data

We then evaluated nOPV1 and nOPV3 immunogenicity in a model requiring efficient replication for induction of antibody responses. Susceptible transgenic mice were infected intraperitoneally with dilutions of Sabin 1, Sabin 3, nOPV1 or nOPV3, and serum samples were collected at 21 days after inoculation and tested by neutralization assay to determine antibody titres (Fig. 4a). Inoculation with Sabin 1 tended to induce higher geometric mean titres of neutralizing antibodies, particularly at low doses (Fig. 4b). However, the difference between antibody titres induced by Sabin 1 and nOPV1 at the tested doses and at the tested sample size did not reach statistical significance, except for low doses (for example, 10^4^ TCID_50_; Fig. 4b). Similarly, we observed no significant difference in antibody titre in mice inoculated with Sabin 3 and nOPV3 (Fig. 4c). Notsly, Sabin 1, Sabin 3, nOPV1 and nOPV3 had similar seroconversion rates and most vaccinated mice generated neutralizing antibodies at the tested doses with a single immunization (Fig. 4b,c, numbers at the top of the graphic). Hence the immunogenicity profiles of nOPV1 and nOPV3 were not significantly inferior to those of Sabin strains.

**Fig. 5:**
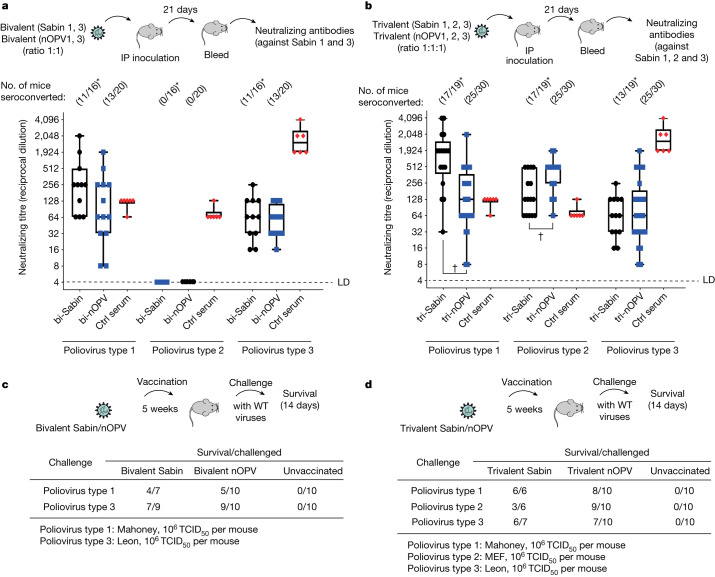
Preclinical efficacy of bivalent and trivalent nOPV in mice with regard to immunogenicity and protection. **a**,**b**, PVRTg21/IFNR-KO mice were inoculated intraperitoneally with 1 × 10^7^ TCID_50_ of a 1:1 mixture of bivalent (**a**) or 1:1:1 mixture of trivalent (**b**) Sabin or nOPV. Either 20 (**a**) or 30 (**b**) mice were used per condition. Asterisks indicate that four bivalent Sabin-immunized mice and 11 trivalent Sabin-immunized mice died and were removed from the experiment. Titres of neutralizing antibodies from mice immunized with bivalent (**a**) or trivalent (**b**) Sabin (black dots) and nOPV (blue squares) against Sabin 1, 2 and 3 were determined. Control (Ctrl) serum was derived from a human participant immunized with OPV (red diamonds). Antibodies from bivalent virus immunized mice were not responsive to type 2 poliovirus (**a**), but shown as the the limit of detection (LD). Box-and-whisker diagrams as in Fig. 4b,c.  †*P* = 0.0001, two-sided Mann–Whitney *U*-test for antibody titres against Sabin 1 and 2 between trivalent Sabin- and trivalent nOPV-immunized serum samples (*n* = 16 and 20, respectively (**a**), *n* = 19 and 30, respectively (**b**) samples). **c**,**d**, Protection of vaccinated mice against pathogenic polioviruses. Mice inoculated with either bivalent formulations (bivalent Sabin or nOPV) (**c**) or trivalent formulations (trivalent Sabin or nOPV) (**d**) were challenged with neurovirulent poliovirus (type 1 Mahoney strain, type 2 MEF strain or type 3 Leon), between 6 and 10 mice per serotype. Data shown as survival over total challenged mice (survival/challenged). WT, wild type. Source Data

Finally, we compared the immunogenicity of the new nOPV vaccine candidates with Sabin strains in bivalent and trivalent formulations. Each Sabin strain (1, 2 and 3) and nOPV1, OPV2 and OPV3 were mixed in either a 1:1:1 (trivalent) or 1:1 (bivalent) proportion of each virus. Mice were inoculated intraperitoneally with 1 × 10^7^ TCID_50_ of the virus of the bivalent and trivalent formulations. We observed similar antibody titres in mice inoculated with Sabin and nOPV bivalent or trivalent formulations (Fig. 5a,b). Furthermore, as with the monovalent formulation, we observed similar seroconversion rates and proportions of seroconverted mice following a single inoculation for Sabin and nOPV bivalent and trivalent formulations (Fig. 5a,b, numbers at the top of the graphic).

We also assessed the correlation between seroconversion and protection in this animal model. Vaccinated mice were challenged with highly pathogenic poliovirus (1 × 10^6^ TCID_50_ of Mahoney type 1, MEF type 2 or Leon type 3, a dose greater than 100 PD_50_) 5 weeks after vaccination. We observed similar levels of protection for Sabin- and nOPV-vaccinated groups after a single vaccination dose (Fig. 5c,d). Interestingly, and in agreement with the lower PD_50_ values and rates of reversion in Sabin strains, several mice immunized with Sabin strains became paralysed or died (Fig. 5a,b, asterisks) but no OPV-vaccinated mice presented any signal of disease.

## Discussion

We describe the development of two new attenuated poliovirus vaccine candidates, nOPV1 and nOPV3. The engineering of these new vaccine candidates was based on the preclinical and clinical information of their parental vaccine strain, nOPV2. Clinical and field information on nOPV2 indicates that its fitness and immunogenicity are similar to Sabin 2 but, importantly, it is genetically more stable than the current Sabin 2 strain. Our original approach was to leverage decades of basic research on the biology of picornaviruses into a rational design of a live attenuated vaccine with improved safety and stability. The five modifications introduced in the Sabin 2 viral genome (Fig. 1a) were based on our understanding of viral biology and pathogenesis. Importantly, because each modification in the nOPVs (*cre5*, S15 *domV*, HiFi(D53N) and Rec1(K38R)) contributes independently to attenuation^18^, their combination in the same virus vaccine strains creates a multilayer safety net that reduces the likelihood of the new Sabin strains regaining fitness and neurovirulence. Accordingly, reversion to a pathogenic virus from the vaccine will require multiple independent mutation events, including several low-probability AU-to-GC double mutations within *domV*, a double recombination event in the 5′ UTR to replace S15 *domV* and preserve *cre5*, and a reversion of the two polymerase modifications that do not reduce replication fitness. A critical feature is that both nOPV1 and nOPV3 maintain their replicative capacity in tissues without causing disease, which is essential to eliciting a robust lifelong immune response. Swapping the coding region of nOPV2 structural proteins with those derived from Sabin 1 and 3 provides protection against the two other poliovirus serotypes, because protection against poliovirus is mediated by neutralizing antibodies directed against epitopes in the capsid proteins^41^.

Notably, none of the modified attenuation or genetic stability determinants map in the nOPV2 capsid region. Thus, replacement of nOPV2 capsid proteins with those from Sabin 1 and 3 should result in viruses with genetic and evolutionary characteristics similar to those of nOPV2 but with immunogenically similar to Sabin 1 and 3 (Fig. 4 and Extended Data Fig. 4). Indeed, in both cell culture (Figs. 1b,c and 2b,c and Extended Data Fig. 1) and small animal models (Figs. 1d and 2d and Extended Data Fig. 2) the concerted action of these modifications produces vaccine candidate strains that, although replicating at similar levels to Sabin 1 and 3, are less virulent than Sabin strains (Fig. 1d and Extended Data Fig. 2) and less likely to evolve into neurovirulent revertants that can become cVDPV strains or cause vaccine-associated paralytic poliomyelitis (Fig. 2 and Extended Data Fig. 3).

In the context of two similar but non-contemporaneous clinical trials, 18–22-week-old infants and 1–5-year-old children were immunized with nOPV2 or Sabin 2. Virus shed from vaccinees was isolated and nucleotide polymorphisms examined by next-generation sequencing^19,23,29,30,39,42^. Also, a considerable amount of sequence data exist for Sabin 2 isolates shed by vaccinees and contacts. Substitutions within the 5′ UTR were present at significant frequencies in viruses excreted by several participants after about 2 weeks of replication, including mutations 215C, 322A, 459C and 498C in domains II, III and IV within the internal ribosome entry site. Mutations 123C or 181G at the base of the *5*′*cre* stem were also observed. These mutations increase the stability of *5*′*cre* by either conversion of a U–G base pair near the foot of the structure into a C–G or the creation of a new C–G base pair at the foot of the stem. Mutation 459C (nucleotide 398 in Sabin 2) was identified as a secondary adaptive mutation in cVDPV2 and vaccinee isolates. Mutations in capsid protein VP1 that increase neurovirulence (VP1-I143T/V) were also observed^1^. Although the frequency of these mutations varied from 7 to 70% among individuals, only a small proportion (13.9%) of shed viruses carried all these mutations^39^. Viruses that had fixed all three modifications—123C or 181G, 459C and I143T/V—were found only in samples taken several weeks post vaccination^19,39^.

The genetic stability and safety of nOPV2 have been documented in preclinical and clinical studies. In the current study, we assessed the genetic stability of nOPV1 and nOPV3 after a ‘forced’ evolution experiment in cell culture (Fig. 2). This experimental strategy best mimics selection pressures in the human gut^43^. Control Sabin 1 and 3 viruses showed increased virulence in a mouse model after ten passages, which correlates with the accumulation of mutations determining neurovirulence. By contrast, we observed no significant increase in the number of paralysed mice at the maximal dose used or accumulation of neurovirulence mutations in nOPV1 and nOPV3 (Fig. 2). This indicates that nOPV1 and nOPV3 have reduced evolutionary rates to neurovirulence. Testing mutations observed in clinical studies also supports the idea that nOPV variants are safer than the original Sabin viruses (Fig. 3c,d). However, continued monitoring of nOPV1/3 evolution would be important because the comprehensive mutational landscapes of nOPV1 and nOPV3 are not available and viruses carrying type 1 and 3 capsid sequences may not take an identical evolutionary trajectory in evolving the same mutations as nOPV2.

The broad use of nOPV2 in millions of children provides support to the concept that the new vaccine is safer than the original Sabin vaccine, with improved genetic stability characteristics^27^. In clinical trials, nOPV2 generated non-inferior immune responses compared with Sabin monovalent OPV2, and all genetic modifications of nOPV2 remained intact^18,23,30,44^. We examined the effects on neurovirulence of mutations emerging during vaccination in humans (Fig. 3a). Even in the worst-case scenario, in which many mutations accumulate in nOPV genomes, the resulting viruses are likely to be much more attenuated than wild-type polioviruses (Fig. 3c,d). Understanding the degree to which this experience is relevant to genetic evolution of nOPV1 and nOPV3 strains will require human clinical studies. Leveraging the results described herein, a phase 1 study comparing the safety, immunogenicity, shedding and genetic stability of nOPV1 and nOPV3 strains with their homotypic Sabin strain OPV controls is ongoing, with primary results anticipated in 2023 (ref. ^45^). Whereas nOPV1 and nOPV3 are currently under development for an outbreak response indication, this intent could be shifted with potential use in supplemental campaigns in high-risk areas or, potentially, even routine use, depending on the epidemiological circumstances.

Finally, bivalent and trivalent nOPV formulations were as immunogenic in mice as the bivalent and trivalent Sabin vaccine (Fig. 5). This finding opens an opportunity to replace multivalent formulations of the Sabin OPV with safer and equally effective nOPV formulations. Because oral live attenuated poliovirus vaccines are required to interrupt person-to-person transmission such as those the world is currently witnessing^13,46^, these improved strains may facilitate the control of poliomyelitis outbreaks around the world.

Whereas nOPV2 is generally deemed to have superior genetic stability compared with Sabin 2, paralysis cases reported in the Democratic Republic of Congo and Burundi and associated with nOPV2-derived viruses^47,48^ signify a need for continued monitoring. Based on the evidence available so far, for nOPV2 to regain wild poliovirus-like virulence it needs to circulate for an extended time, during which it must mutate and recombine with related unattenuated viruses. It is important to note, however, that such events are infrequent and have not been observed in clinical studies. Low vaccine coverage during outbreak response campaigns or low type 2 immunity in adjacent populations increases the risk of such events, regardless of OPV2 choice.

## Methods

### Experimental model and participant details

#### Cells and viruses

HEp2C (NIBSC 740502), HeLa S3 (ATCC CCL-2.2) and Vero (ATCC CCL-81) cells were obtained from either NIBSC or ATCC for this study. Vero cells were maintained in Eagle’s minimum essential medium supplemented with 10% fetal bovine serum (Sigma-Aldrich) and 1× penicillin/streptomycin (Invitrogen) at 37 °C with 5% CO_2_. Mycoplasma was not detected and no signs of contamination were observed. HEp2C and HeLa S3 cells were maintained as previously described^49^. All viruses were generated from infectious cDNA clones and propagated and titred by TCID_50_ assay as previously described^49^.

### Method details

#### Construction of nOPV1 and nOPV3

DNA fragments, synthesized de novo and including the entire capsid coding regions of Sabin 1 and 3 and the downstream nOPV2 2A sequence up to the BseRI site (3888), were introduced into the nOPV2 infectious clone^16^ using SacI and BseRI sites. As a result, the capsid coding sequence of nOPV1 was identical to that of Sabin 1 except for a silent A–T mutation at nucleotide 817 creating a SacI restriction site; the capsid coding sequence of nOPV3 was identical to that of Sabin 3 and the remainder of each genome was identical to nOPV2.

#### Generation of viruses from infectious cDNA clones

Viruses were generated as previously described^49^. In brief, 20 μg of in vitro transcribed viral RNA was electroporated into 4 × 10^6^ HeLa S3 cells. Electroporated cells were incubated at 33 °C for 24 h and harvested as P0. Sanger sequencing and either a standard TCID_50_ or plaque assay were performed to confirm the sequence and determine virus titre. HeLa S3 cells were infected with P0 virus at MOI = 0.1, incubated at 33 °C for 24 h and then harvested as P1. P1 virus was used for all experiments unless noted otherwise. All viruses inoculated into mice were analysed by next-generation sequencing. No polymorphisms with frequencies of over 0.5% were detected in P1 viruses.

#### Virus replication analysis

One-step replication analysis was performed as previously described^49^ and cells were incubated at 33 °C. Virus yield was evaluated in triplicate, with Vero cells preseeded onto six-well plates (10^6^ cells per well) the day before the experiment. Briefly, preseeded cells were infected at MOI = 0.01 and allowed to stand at room temperature, with gentle agitation every 15 min, for 1 h to facilitate virus adsorption. After one PBS wash, viral medium was added to each well and the infected cells incubated at 33.0, 37.0 or 39.5 °C for 48 h. Virus was harvested by three freeze–thaw cycles and clarified by centrifugation. Virus titres were determined by a standard TCID_50_ assay and calculated with the Spearman–Karber method^50^.

#### Genetic stability after accelerated evolution

The genetic stability of Sabin 1/3 and nOPV1/3 was evaluated by deep sequencing of virus populations before and after accelerated evolution. In brief, Vero cells were infected with virus at MOI = 0.1 and incubated at 37 °C to accelerate virus evolution. Virus populations were harvested after 10 h and titred with a TCID_50_ assay for the following passage. After ten passages under accelerated evolution, viral RNA was isolated using the ZR Viral RNA kit (Zymo Research) following the manufacturer’s protocol. The KAPA Stranded mRNA-Seq kit (KAPA Biosystems) and NEXTflex RNA sequencing (RNA-seq) barcodes were used to prepare RNA libraries for sequencing, as previously described^18^. Library fragment size distribution was determined using a Bioanalyzer High Sensitivity DNA Assay (Agilent) and concentrations determined using the KAPA Library Quantification Kit for Illumina Platform (KAPA Biosystems). Deep sequencing was performed using MiSeq (Illumina), producing 300 nt paired-end reads. Sequencing reads were analysed using Lowfreq to determine variant frequency^51^.

#### Animal experiments

PVRTg21/IFNR-KO mice were used for immunogenicity assay. The mouse strain was provided by S. Koike and maintained in an AAALAC-certified animal facility at UCSF. All procedures were performed according to the guidelines of the Laboratory Animal Center of the National Institutes of Health. The Institutional Animal Care and Use Committee of the University of California, San Francisco approved all animal protocols (Approved protocol no. AN194006-01A).

#### Virulence evaluation

Ten-day-old PVRTg21/IFNR-KO mice were inoculated intraperitoneally with 100 μl of inoculum delivering various doses (10^3^–10^7^ TCID_50_ of virus) and survival was monitored for 21 days. Mice were euthanized and marked dead when the humane endpoint (paralysis of both posterior limbs) was observed.

#### Immunogenicity assay

Four-week-old PVRTg21/IFNR-KO mice were randomly allocated to groups for intraperitoneal injection of 100 µl of inoculum delivering various doses (10^7^–10^4^ plaque-forming units of virus per mouse, ten mice per dose) of Sabin 1/3 or nOPV1/3. Blinding was not applied. Blood samples were collected from the retro-orbital sinus on day 21 post inoculation for neutralization assay. Following inactivation at 55 °C for 30 min, a series of twofold serial dilutions of serum was prepared and mixed with 100 TCID_50_ of poliovirus virus and maintained at 37 °C for 2 h. The virus–serum mixture was then transferred to another 96-well plate preseeded with HeLa S3 cells (10^4^ per well) and incubated at 33 °C for 7 days. The plates were fixed with formaldehyde and stained with 0.5% crystal violet to determine cytopathic effect. Antibody titre was defined as the highest dilution sufficient to inhibit the development of cytopathic effect. Pre-immunized human serum was included as a neutralizing standard serum. The starting dilution was fourfold, and serum samples showing no inhibition at the starting dilution were excluded from the graph.

#### TgmNVT

The potential neurovirulence of nOPVs was assessed in human poliovirus receptor transgenic mice using a method adapted from the standard operating procedure “WHO neurovirulence test of type 2 live poliomyelitis vaccines (oral) in transgenic mice susceptible to poliovirus” available from the WHO^36^; the adaptation of the standard operating procedure included the use of higher doses and fewer mice per dose. Additionally, Tg66 mice (which also express the human poliovirus receptor)^52^ were used as a substitute for the TgPVR21 strain used in the WHO assay. Both mouse strains have a sensitivity similar to that of Sabin 2 when inoculated by the intraspinal route. In brief, 6–8-week-old mice in groups of eight (weight and sex matched) were sedated and inoculated in the lumbar region of the spinal cord with 5 µl of each dose and observed for signs of paralysis for up to 14 days. Mice with paresis/paralysis were scored positive, and those surviving for 14 days with no clinical signs were scored negative. When possible, PD_50_ was calculated using the Spearman–Karber method.

The TgPVR mouse experiments at NIBSC were performed under licence nos. PPL 80/2478 and PPL 70/8979, granted by the UK Home Office under the Animal (Scientific Procedures) Act 1986, revised 2013, and reviewed by the internal NIBSC Animal Welfare and Ethics Review Board before submission.

#### Antigenicity assay

A non-competitive sandwich ELISA assay^53^ was used to measure reactivity with monoclonal antibodies specific for four different antigenic sites present on native virus particles. Briefly, twofold dilutions of antigen were captured with a serotype-specific polyclonal antibody and then detected using serotype-specific monoclonal antibodies and anti-mouse peroxidase conjugate. The reactivity of each test sample was evaluated against a Sabin 1 or 3 reference. Inactivated polio vaccine was included as a control.

#### Statistical analyses

The data below were prepared and analysed with Prism 8 (GraphPad) using statistical methods described below. Statistical significance was set as *P* < 0.05. All multiple test correction was performed using the Holm–Sidak method^54^ and is reported below as corrected *P* value.

In Fig. 1b, data are mean ± s.d. of triplicates. Student’s *t*-tests were used to compare titres of type 1 or 3 viruses each time, with correction for multiple comparisons. The analysis suggested no significance at the tested time points. In Fig. 1c, Student’s *t-*tests were used to compare the virus yield of Sabin 1 and nOPV1, and of Sabin 3 and nOPV3, at the tested temperatures. For type 1 polioviruses, *P* values were 0.1202, 0.7076 and 0.000002 for 33, 35 and 37 °C, respectively. For type 3 polioviruses, *P* values were 0.5110, 0.0819 and 0.1441 for 33, 35 and 37 °C, respectively.

In Fig. 4, data are presented as box-and-whisker plots. The Mann–Whitney *U*-test was performed to compare antibody titres induced by Sabin 1 and nOPV1 (Fig. 4b) and by Sabin 3 and nOPV3 (Fig. 4c). Differences in titres of the induced antibodies between Sabin 1 and nOPV1 did not reach statistical significance at higher doses (*P* = 0.6296, 0.6706, and 0.0622 for 10^7^, 10^6^, and 10^5^ TCID_50_, respectively), but a significant difference was observed at the lowest dose (0.0225 for 10^4^ TCID_50_ of virus). For type 3 viruses, the difference in titres of the induced antibodies did not reach significance at the tested doses (*P* = 0.2591, 0.3598, 0.4960 and 0.1127 for 10^7^, 10^6^, 10^5^ and 10^4^, respectively).

In Fig. 5a,b, data are presented as box-and-whisker plots. A Mann–Whitney *U*-test was performed to compare antibody titres induced by the bivalent (Fig. 5a) or trivalent (Fig. 5b) virus. The difference in titres of neutralizing antibodies induced by bivalent Sabin 1/3 and nOPV1/3 did not reach significance (*P* = 0.1073 and 0.7072 for types 1 and 3, respectively). The difference in neutralizing titres induced by trivalent Sabin 1/3 and nOPV1/3 reached significance against poliovirus type 1 (*P* = 0.0001) and type 2 (*P* = 0.0041), but not against type 3 (*P* = 0.6694).

### Reporting summary

Further information on research design is available in the Nature Portfolio Reporting Summary linked to this article.

## Online content

Any methods, additional references, Nature Portfolio reporting summaries, source data, extended data, supplementary information, acknowledgements, peer review information; details of author contributions and competing interests; and statements of data and code availability are available at 10.1038/s41586-023-06212-3.

## Supplementary information

Reporting Summary

## Data Availability

Sequencing data can be accessed on the SRA database, accession no. PRJNA951077. All data are available in the main text or the Extended Data materials. Further information and requests for resources and reagents should be directed to, and will be fulfilled by, the lead contacts (Andrew.Macadam@nibsc.org and raul.andino@ucsf.edu). Source data are provided with this paper.

## Source data

Source Data Fig. 1
Source Data Fig. 2
Source Data Fig. 3
Source Data Fig. 4
Source Data Fig. 5
Source Data Extended Data Fig. 1
Source Data Extended Data Fig. 2
Source Data Extended Data Fig. 4

## Code availability

RNA-seq data were analysed using LoFreq^51^, which is available on the developers’ website (https://csb5.github.io/lofreq/). No other custom code was used in this study.
