# Supplementary material for: Genetic stabilization of attenuated oral vaccines against poliovirus types 1 and 3
Source: Nature. 2023 Jun 14;619(7968):135–42. doi: 10.1038/s41586-023-06212-3 (PMC10322712; doi:10.1038/s41586-023-06212-3)
Supplement: Supplementary file 1 — Reporting Summary [file 41586_2023_6212_MOESM1_ESM.pdf]

nature portfolio

Corresponding author(s): Andrew Macadam
Raul Andino

Last updated by author(s): Apr 12, 2023

Reporting Summary

Nature Portfolio wishes to improve the reproducibility of the work that we publish. This form provides structure for consistency and transparency in reporting. For further information on Nature Portfolio policies, see our Editorial Policies and the Editorial Policy Checklist.

Statistics

For all statistical analyses, confirm that the following items are present in the figure legend, table legend, main text, or Methods section.

n/a	Confirmed	
☐	☒	The exact sample size (*n*) for each experimental group/condition, given as a discrete number and unit of measurement
☐	☒	A statement on whether measurements were taken from distinct samples or whether the same sample was measured repeatedly
☐	☒	The statistical test(s) used AND whether they are one- or two-sided<br>*Only common tests should be described solely by name; describe more complex techniques in the Methods section.*
☒	☐	A description of all covariates tested
☐	☒	A description of any assumptions or corrections, such as tests of normality and adjustment for multiple comparisons
☐	☒	A full description of the statistical parameters including central tendency (e.g. means) or other basic estimates (e.g. regression coefficient) AND variation (e.g. standard deviation) or associated estimates of uncertainty (e.g. confidence intervals)
☐	☒	For null hypothesis testing, the test statistic (e.g. *F*, *t*, *r*) with confidence intervals, effect sizes, degrees of freedom and *P* value noted<br>*Give P values as exact values whenever suitable.*
☒	☐	For Bayesian analysis, information on the choice of priors and Markov chain Monte Carlo settings
☒	☐	For hierarchical and complex designs, identification of the appropriate level for tests and full reporting of outcomes
☒	☐	Estimates of effect sizes (e.g. Cohen's *d*, Pearson's *r*), indicating how they were calculated

Our web collection on statistics for biologists contains articles on many of the points above.

Software and code

Policy information about availability of computer code

Data collection	RNAseq was performed on Miseq platform (Illumina).
Data analysis	Data was analyzed and graphed with GraphPad Prism Version 8. RNAseq data was analyzed with LoFreq (DOI: 10.1093/nar/gks918), available at https://csb5.github.io/lofreq/

For manuscripts utilizing custom algorithms or software that are central to the research but not yet described in published literature, software must be made available to editors and reviewers. We strongly encourage code deposition in a community repository (e.g. GitHub). See the Nature Portfolio guidelines for submitting code & software for further information.

Data

Policy information about availability of data

All manuscripts must include a data availability statement. This statement should provide the following information, where applicable:

- Accession codes, unique identifiers, or web links for publicly available datasets
- A description of any restrictions on data availability
- For clinical datasets or third party data, please ensure that the statement adheres to our policy

Data availability statement has been included in the manuscript as below.
Sequencing data can be accessed on the SRA database (Accession number: PRJNA951077). All data are available in the main text or the Extended Data materials.

Further information and requests for resources and reagents should be directed to and will be fulfilled by the Lead Contacts Andrew.Macadam@nibsc.org; raul.andino@ucsf.edu

Human research participants

Policy information about studies involving human research participants and Sex and Gender in Research.

Reporting on sex and gender	N/A
Population characteristics	N/A
Recruitment	N/A
Ethics oversight	N/A

Note that full information on the approval of the study protocol must also be provided in the manuscript.

Field-specific reporting

Please select the one below that is the best fit for your research. If you are not sure, read the appropriate sections before making your selection.

☒ Life sciences ☐ Behavioural & social sciences ☐ Ecological, evolutionary & environmental sciences

For a reference copy of the document with all sections, see nature.com/documents/nr-reporting-summary-flat.pdf

Life sciences study design

All studies must disclose on these points even when the disclosure is negative.

Sample size	Sample size was not predetermined by statistical methods. For virus replication analysis, the number of replicates is 3, which was chosen as the minimal replicate number to enable statistical analysis. For animal experiments, both male and female mice were used and the sample size (8-10 per condition) was based on our previous study (Yeh et. al., Engineering the Live-Attenuated Polio Vaccine to Prevent Reversion to Virulence. Cell Host & Microbes. 27, 2020).
Data exclusions	No data was excluded.
Replication	For mouse experiment, each condition was tested with 8-10 mice for survival or antibody response. Data correspond to individual mouse, so replication in duplicate or triplicate was not applied. At least two independent experiments were performed and all attempts were successful. For virus growth analysis, virus infection was performed in triplicates for each collected condition. At least two independent experiments were performed and all attempts were successful.
Randomization	Animals and cells were randomly allocated to experimental groups.
Blinding	Blinding was not applied as results are not subject to researchers' bias.

Reporting for specific materials, systems and methods

We require information from authors about some types of materials, experimental systems and methods used in many studies. Here, indicate whether each material, system or method listed is relevant to your study. If you are not sure if a list item applies to your research, read the appropriate section before selecting a response.

Materials & experimental systems

n/a	Involved in the study	
☐	☒	Antibodies
☐	☒	Eukaryotic cell lines
☒	☐	Palaeontology and archaeology
☐	☒	Animals and other organisms
☒	☐	Clinical data
☒	☐	Dual use research of concern

Methods

n/a	Involved in the study	
☒	☐	ChIP-seq
☒	☐	Flow cytometry
☒	☐	MRI-based neuroimaging

Antibodies

Antibodies used

Monoclonal antibodies used in this study were in-house generated as described (Minor, P. D., Ferguson, M., Evans, D. M. A., Almond, J. W. & Icenogle, J. P. Antigenic Structure of Polioviruses of Serotypes 1, 2 and 3. J Gen Virol 67, 1283–1291 (1986)).

monoclonal antibodies (MAbs), specific for Sabin1 native conformations of antigenic sites 1 (MAb 955), 2 (MAbs 237), 3 (MAb 424). and 4 (MAbs 234); specific for Sabin3 native conformations of antigenic site 1 (MAb 520), 2 (MAbs 877), 3 (MAb 883), and 4 (MAbs 1281). These antibodies were prepared in a 2-fold serial dilution for an antigenicity assay as described in the Method section, so no specific dilution was applied.

anti-mouse conjugate: goat-anti-mouse IgG secondary antibody, HRP (Invitrogen 62-6520); dilution: 1:3000

Validation

These in-house generated antibodies are previously described (Minor, P. D., Ferguson, M., Evans, D. M. A., Almond, J. W. & Icenogle, J. P. Antigenic Structure of Polioviruses of Serotypes 1, 2 and 3. J Gen Virol 67, 1283–1291 (1986).)

Eukaryotic cell lines

Policy information about cell lines and Sex and Gender in Research

Cell line source(s)

Hep2C (NIBSC 740502)
HeLa S3 (ATCC CCL-2.2)
Vero (ATCC CCL-81)

Authentication

These cells were obtained from NIBSC or ATCC, we didn't perform validation.

Mycoplasma contamination

Cell lines were not tested for mycoplasma contamination, but no signs of contamination was observed.

Commonly misidentified lines
(See ICLAC register)

None.

Animals and other research organisms

Policy information about studies involving animals; ARRIVE guidelines recommended for reporting animal research, and Sex and Gender in Research

Laboratory animals

Four-week-old PVRTg21 mice were used for immunogenicity assay,
Six-eight-week-old PVRTg66 and 10-day-old PVRTg21/IFNR-ko mice were used for virulence test.
All mice were housed in specific pathogen-free facilities under a 12:12 h light:dark cycle with a temperature of 25°C and humidity of 35%.

Wild animals

No wild animals were used in this study.

Reporting on sex

For experiments with adult mice, equal number of male and female mice were included in each experimental group.
For experiments with 10-day-old mice, the entire liters were used, as these newborn mice were too young to be weaned.

Field-collected samples

No field-collected samples were used in this study.

Ethics oversight

UCSF: The Institutional Animal Care and Use Committee of the University of California, San Francisco approved all animal protocols (Approved protocol No. AN194006-01A).
NIBSC: Mouse experiments at NIBSC were performed under licenses PPL 80/2478 and PPL 70/8979 granted by the UK Home Office under the Animal (Scientific Procedures) Act 1986 revised 2013 and reviewed by the internal NIBSC Animal Welfare and Ethics Review Board before submission.

Note that full information on the approval of the study protocol must also be provided in the manuscript.
